# Rickets as a Dietetic Disease

**Published:** 1919-04-05

**Authors:** 


### RICKETS AS A DIETETIC DISEASE.

### An Outline of Recent Research.

In a recent issue of this journal was published a note upon the work and future requirements of the Medical Research Committee, and a brief outline is now given of one of the many valuable investigations undertaken for this body. The experimental work has been largely carried out by Dr. Edward Mellanby, and described by him in lectures at the Royal College of Surgeons. The results, though not claimed as final, throw considerable light upon the essential causes of one of our greatest social evils.

The European range and frequency of rickets have long been realised, but it is doubtful if the indirect influences of this disease have met with adequate appreciation. Taking an average from a large number of examinations of children in towns and densely populated areas, it is found that over seventy-five per cent. show some evidence of the condition. One of the greatest dangers constantly menacing the health of civilised communities, namely the future development of defective and carious teeth, has been shown to bear a very definite relation to the rachitic condition, and, if the results be followed further, it is seen that the markedly reduced resistance of the rachitic child may not only account to a large extent for the enormous loss of infant life in poor urban districts of this country, but also predispose in later youth to fatal terminations for normally subacute illness or to infection by organisms such as tubercle bacilli or low-grade streptococci, against which the healthy body has considerable powers of resistance. It is more than probable that, although not in itself a fatal disease, rickets may be one of the most potent factors in the relatively high death-rate among all town-dwellers.

### The Anti-rachitic Factor.

Dr. Mellanby's experiments were performed with a series of puppies five to eight weeks old. In the first place it was necessary to determine exactly what diets could be expected consistently to produce rickets and at the same time keep the animals in sufficiently good health to avoid a high mortality from marasmus or bronchopneumonia. A satisfactory routine having been devised, a fresh set of puppies were started, with various substances added to their daily food, and their resulting development carefully noted. Records were made of the CaO content of their bones and the presence or absence of disease confirmed in every case by histological methods.

The definitely rachitic diets were made up as follows:—

No. I.—Whole milk, 175c.c.; oatmeal, rice and 1.2g. NaCl.

No. II.—Whole milk, 175c.c.; bread *ad lib.*

No. III.—Separated milk, 175c.c.; bread (70 per cent. wheaten) *ad lib.*; linseed oil, 10c.c.; yeast, 10g.; NaCl, 2g.

No. IV.—Separated milk, 250-230c.c.; bread (70 per cent. wheaten) *ad lib.*; linseed oil, 5-15c.c.; yeast, 5-10g.; orange juice, 3c.c.; NaCl, 1-2g.

Using I., an increase of whole milk up to 500c.c. was found to prevent disease and with II., meat or its watery and alcoholic extracts had a strong inhibitory effect, but the protein residue of meat after the removal of all extractives allowed marked rachitic development. Yeast had no protective influence, but malt extract slightly delayed the onset. With III., a diet in which separated milk free from milk fat was used a comparative observation was made by adding different common fats and oils to the meals and a definite gradation of anti-rachite powers obtained. Cod liver oil and butter were found satisfactory and linseed oil useless, in fact all vegetable fats were far inferior to those of animal origin. The addition of orange juice had no preventive effect, while 5g. of calcium phosphate or doubling the quantity of separated milk to thus increase the calcium intake were both without beneficial result.

All these observations suggest that rickets is a deficiency disease and develops in the absence of some essential food factor. Three such elements have been recognised by authorities and one may, in lack of more descriptive terms, name them fat-soluble A, water-soluble B, and the anti-scorbutic. The inefficiency of yeast and orange juice dispose of any claims for the last two types and, although it is at present impossible to say definitely that the anti-rachite factor and the fat-soluble A of other workers are identical, yet Dr. Mellanby considers that, in view of their remarkably similar distribution, a very close relationship is more than probable.

### Compatibility with Earlier Theories.

In England firmly rooted opinions originated with the successful experiments of Bland-Sutton upon the lion cubs in Regent's Park. Additions of milk, pounded bone, and cod-liver oil to their meat diet caused the immediate disappearance of rickets in these animals, and for the first time the society succeeded in rearing the young. Also the established and beneficial results of administration of cod-liver oil to children has led fat deficiency to be widely considered as the primary cause of rickets, but these experiments prove that this good depends entirely upon which fat is utilised; certain types are greatly superior to others.

Cereals treated by modern manufacturing processes are found markedly wanting in preventive effect. The view that disease originates from an excess of carbohydrate in the diet is possibly true, but not owing to the direct effect of carbohydrate itself. Meals containing a large amount of cereal tend by bulk, and the effect on appetite alone automatically to reduce the intake of both fat and many substances known to be strongly beneficial. Also the theory regarding deficiency of calcium salts in the food is largely disproved. The direct addition

**Rickets as a Dietetic Disease**—(*continued*).

of calcium to a rachitic diet is seen above to have no favourable influence upon the result, and it is stated that, if the rachitic factor in a diet be sufficiently strong, then considerable reduction in the calcium content completely fails to produce rickets. Probably lack of these salts tends, when accompanied by a factor-deficient diet, to give rise to an unusually severe form of the disease, but does not in itself constitute an original cause.

The Influence of Social Conditions.

With regard to the idea that the condition was produced by the unsatisfactory hygiene of crowded communities, one feels that the experimental work itself points so strongly to a dietetic origin that, although undoubtedly present-day tenement life predisposes to a condition of low grade health and bodily tone in children, the blame for the onset of rickets cannot be rightly attached to surroundings alone. A few years ago Leonard Findlay, by investigations conducted on a much smaller scale than those here reviewed, suggested that confinement and lack of exercise were a primary cause. His dogs were fed upon oatmeal, porridge, and milk, but the amount of the last-named was indefinite, and although this diet approximates to No. I., the variable amount of milk makes it not strictly rachitic. His confined dogs certainly developed rickets, but it must be remembered that exercise or the freedom to obtain it improves health on even an inadequate diet, and also it may be assumed that a rachitic diet is more likely to produce signs of disease when the bodily health is kept constantly subnormal by surroundings. The marked characteristic of the rickety child is its disinclination to make any large amount of voluntary movement whatever degree of freedom may be granted to it. Dr. Mellanby states that in his own experience no amount of confinement will produce the disease in animals if they are adequately fed on diets containing the suggested anti-rachitic factor in sufficient quantity.

The Value of Common Foods.

The more usual articles of food are next classified. Among those deficient in the essential property are found flour, potatoes, sugar, oatmeal, and other cereals, while those of definite preventive power are milk, meat, margarine or butter, fish, eggs, and cheese, namely the "good things" of the table. Statistics have been drawn up which show that, to some extent, there is a slight predominence of rachitic substances in the average diets of families showing a tendency to the disease, but the absolute deficiency of the anti-rachitic element is not so great as might be expected. A very possible explanation of this lies, however, in the fact that the parents of these families are, with noticeable frequency, of the careless type, and in that case the younger children would quite probably fail to receive their fair share of the good things, and be given an undue proportion of the common ricket-producing foodstuffs.

In any case it seems proved that there is some food factor of which the infant must receive the necessary quantity, and that it exists in the articles named. To the absolute amount of food which a child can take there is obviously a limit, and the diet question therefore partially depends on the balance of constituents, and we must consider neutral substances if occurring to too high proportion as actually ricket-producing. Excess of bread and cereals are the worst offenders in crowding out the more necessary items. The nature of fat given is of the greatest importance, and a child should not be fed on vegetable margarine or any form of vegetable fat unless a satisfactory amount of butter or animal products can also be provided. If accessory fat be needed, cod-liver oil is by far the best.

Dr. Mellanby draws attention to the fact that, although milk should be considered the necessary and staple article of a child's food for several years after birth, there may on occasions be some exception to this. It has been shown that fat-soluble A is slightly, if at all, synthesised in the body of the female during lactation, and must be sufficiently present in her diet if the milk is to transmit the factor. In the case of the cow, green grass amply supplies this need, but in winter months the artificial diet of these animals, *i.e.*, oil-cake, etc., is frequently deficient, and this circumstance may account for both unexpected and seasonal incidence of the disease.

General Applications.

On the whole, there is a strong case made for the dietetic theory, and the experimental results are sufficiently definite and convincing to suggest that a careful survey of all proprietary articles of food for children should be made with a view to determining not the customary analysis as to the quantity of protein, fat, carbohydrates and salts they contain—here a relatively useless procedure—but the determination of the exact nature of the contents, and therefore the proportion food factors.

Past views that rickets was strictly a dietetic disease, and that good food will prevent its onset, have been true in a broad sense, but in this recent research we see that the first steps towards recognition of an essential food factor, parallel to the anti-scorbutic, have been made. Even in the groups into which constituents of diet have been formerly divided, we find the individual members varying to extreme degrees of relative factor content, and therefore must we not only reset our picture of the disease itself, but also in future considerably revise our preventive measures and advice.

